# Low-temperature at booting reduces starch content and yield of wheat by affecting dry matter transportation and starch synthesis

**DOI:** 10.3389/fpls.2023.1207518

**Published:** 2023-06-14

**Authors:** Wenjing Zhang, Anmin Zhang, Qirui Zhou, Ranran Fang, Yan Zhao, Zihong Li, Jiawen Zhao, Mengting Zhao, Shangyu Ma, Yonghui Fan, Zhenglai Huang

**Affiliations:** ^1^Key Laboratory of Wheat Biology and Genetic Improvement on South Yellow and Huai River Valley, The Ministry of Agriculture, Hefei, Anhui, China; ^2^Department of Agronomy, Anhui Agricultural University, Hefei, Anhui, China

**Keywords:** wheat, low-temperature stress, booting, dry matter transportation, starch synthesis

## Abstract

With the continuous change of global climate, the frequency of low-temperature stress (LTS) in spring increased greatly, which led to the increase of wheat yield decline. The effects of LTS at booting on grain starch synthesis and yield were examined in two wheat varieties with differing low-temperature sensitivities (insensitive variety Yannong 19 and sensitive variety Wanmai 52). A combination of potted and field planting was employed. For LTS treatment at booting, the wheat plants were placed in a climate chamber for 24 h at −2°C, 0°C or 2°C from 19:00 to 07:00 then 5°C from 07:00 to 19:00. They were then returned to the experimental field. The effects of flag leaf photosynthetic characteristics, the accumulation and distribution of photosynthetic products, enzyme activity related to starch synthesis and relative expression, the starch content, and grain yield were determined. LTS at booting caused a significant reduction in the net photosynthetic rate (*P*_n_), stomatal conductance (*G*_s_), and transpiration rate (*T*_r_) of the flag leaves at filling. The development of starch grains in the endosperm is also hindere, there are obvious equatorial grooves observed on the surface of the A-type starch granules, and a reduction in the number of B-type starch granules. The abundance of ^13^C in the flag leaves and grains decreased significantly. LTS also caused a significant reduction in translocation amount of pre-anthesis stored dry matte from vegetative organs to grains and amount of post-anthesis transfer of accumulated dry matte into grains, and the distribution rate of dry matter in the grains at maturity. The grain filling time was shortened, and the grain filling rate decreased. A decrease in the activity and relative expression of enzymes related to starch synthesis was also observed, with a decrease in the total starch content. As a result, a decrease in the grain number per panicle and 1000-grain weight were also observed. These findings highlight the underlying physiological cause of decreased starch content and grain weight after LTS in wheat.

## 1 Introduction

LTS in spring is one of the main limiting factors affecting wheat production. The frequent occurrence of extreme weather events and climate warming is causing early onset of the wheat phenological period, aggravating low-temperature damage in spring (Trnka et al., 2014). In the Huanghuai region of China, LTS in spring lasts for a long duration and is of high intensity, causing serious wheat yield losses (Xiao et al., 2018). These low-temperatures typically occur from the end of March to the beginning of April, and although the young wheat panicle remains enclosed in the flag leaf sheath at this time, it represents a critical and sensitive stage of meiosis and tetrad formation. LTS affects wheat growth and development (Kumar et al., 2018), reducing the photosynthetic capacity of functional leaves (Gupta et al., 2016) and causing a decrease in the carbohydrate content (Shahryar and Maali-Amiri, 2016), ultimately leading to a decrease in yield (Obembe et al., 2021).

Photosynthesis is one of the basic processes to ensure stress of plants (Kreslavski et al., 2013). Studies have shown that LTS leads to a decrease in *P*_n_, *G*_s_, and *T*_r_ in wheat (Yordanova and Popova, 2007). Meanwhile, other studies have shown a reduction in photosynthetic capacity under stress, as well as a decrease in glycogen supply to source organs and subsequent transportation to sink organs (Clusters et al., 2016), which in turn affect the accumulation and distribution of dry matter. Drought stress was also found to cause a decrease in dry matter distribution in wheat grains (Dhakal, 2021), while waterlogging stress caused a 57% reduction in post-anthesis dry matter accumulation (Palta et al., 1994).

Starch is the main component of the wheat endosperm, and is synthesized via a series of coordinated enzymatic reactions (Panigrahi et al., 2019). Starch phosphorylase (Pho1, EC 2.4.1.1) is a temperature-dependent enzyme, the main role of which is the regulation of starch synthesis at low-temperature (Satoh et al., 2008). Recent studies have also revealed a role of disproportionating enzyme (Dpe1, EC 2.4.1.25) in starch synthesis (Dong et al., 2015; Vinje et al., 2022). Moreover, Dpe1 was found to participate in starch structure modification by transferring maltose to amylopectin, with over-expression resulting in small starch granules, and inhibition an increase in amylose content (Dong et al., 2015).

Moreover, both low- and high-temperature stress during grain filling resulted in a reduction in the grain filling rate of wheat, affecting the synthesis of amylopectin and total starch, and in turn causing reductions in the grain starch content and weight (Zhao et al., 2022). LTS resulted in a reduction in grain size and plumpness during grain filling (Cromey et al., 1998), and a reduction in grain number per spike and the 1000-grain weight (Kajla et al., 2015; Zhang et al., 2021b).

Previously, it has been reported that LTS at booting will adversely affect wheat yield, and wheat grain development mainly depends on the process of starch synthesis and accumulation (Liu et al., 2019; Xiao et al., 2022). Therefore, studying the effect of LTS at booting on grain weight formation is necessary. Although numerous reports have documented the effects of LTS in spring on photosynthetic characteristics and yield in wheat, few reports have examined the effect of LTS at booting on grain starch content and yield from the perspective of dry matter transportation and starch synthesis. In this study, we selected the varieties whose yield and its components decreased slightly and greatly in the spring LTS test of wheat varieties planted in a large area in this area as the experimental materials (the data has not been published yet). And wheat varieties with differing low-temperature sensitivities were subjected to LTS at booting. Photosynthetic characteristics of flag leaves, the accumulation and transportation of photosynthetic products, the activity and relative expression of starch synthesis related enzymes in grains, the starch content and yield in grains were determined. The findings provide a physiological basis for the reduction in starch content and grain weight resulting from LTS at booting.

## 2 Materials and methods

### 2.1 Plant materials

Two wheat varieties with differing sensitivities to LTS were selected based on the degree of decrease of grain number per spike and grain weight: insensitive variety Yannong 19 (bred by the Wheat Research Institute, Yantai Academy of Agricultural Sciences, Shandong Province, China) and sensitive variety Wanmai 52 (bred by Suzhou Seed Company, Anhui Province, China).

### 2.2 Experimental design

The experiments were carried out at the on-campus experimental base of Anhui Agricultural University (Hefei, Anhui Province, China; 31.52°N, 117.17°E) from November 2018 to June 2019, and November 2020 to June 2021. Seeds were sown on 6 November 2018 and 5 November 2020, respectively. The experiments used a combination of potted and field planting methods. Pots were 30 cm high and 30 cm in diameter, and were potted with soil taken from the 0–20 cm layer of the experimental field. The nutrient content of the experimental field before sowing is shown in **Table 1**. Each pot was filled with 10 kg of sifted soil plus 75 g of organic fertilizer, 8 g of compound fertilizer (N: P: K = 17: 17: 17), and 4.91 g of urea. An additional 2.28 g of urea was added to each pot as topdressing at jointing. A total of 120 pots were planted per variety. They were then buried in the experimental field, with the upper edge of the pots flush with the ground. Emerging seedlings showing uniform growth were thinned to 10 seedlings per pot. All other field management measures were in accordance with the requirements of high-yield cultivation.

**Table 1:** The nutrient content of the experimental field before sowing.

Year	Organic matter (g·kg^−1^)	Total N (g·kg^−1^)	Available N (mg·kg^−1^)	Available P (mg·kg^−1^)	Available K (mg·kg^−1^)	pH
2018—2019	14.8	1.09	90.6	16.5	79.6	6.15
2020—2021	17.4	0.91	87.5	14.8	80.7	6.19

Before sowing, the soil in the experimental field was sampled, and the related indexes of the soil were measured and analyzed in the laboratory environment.

**Figure 1:**
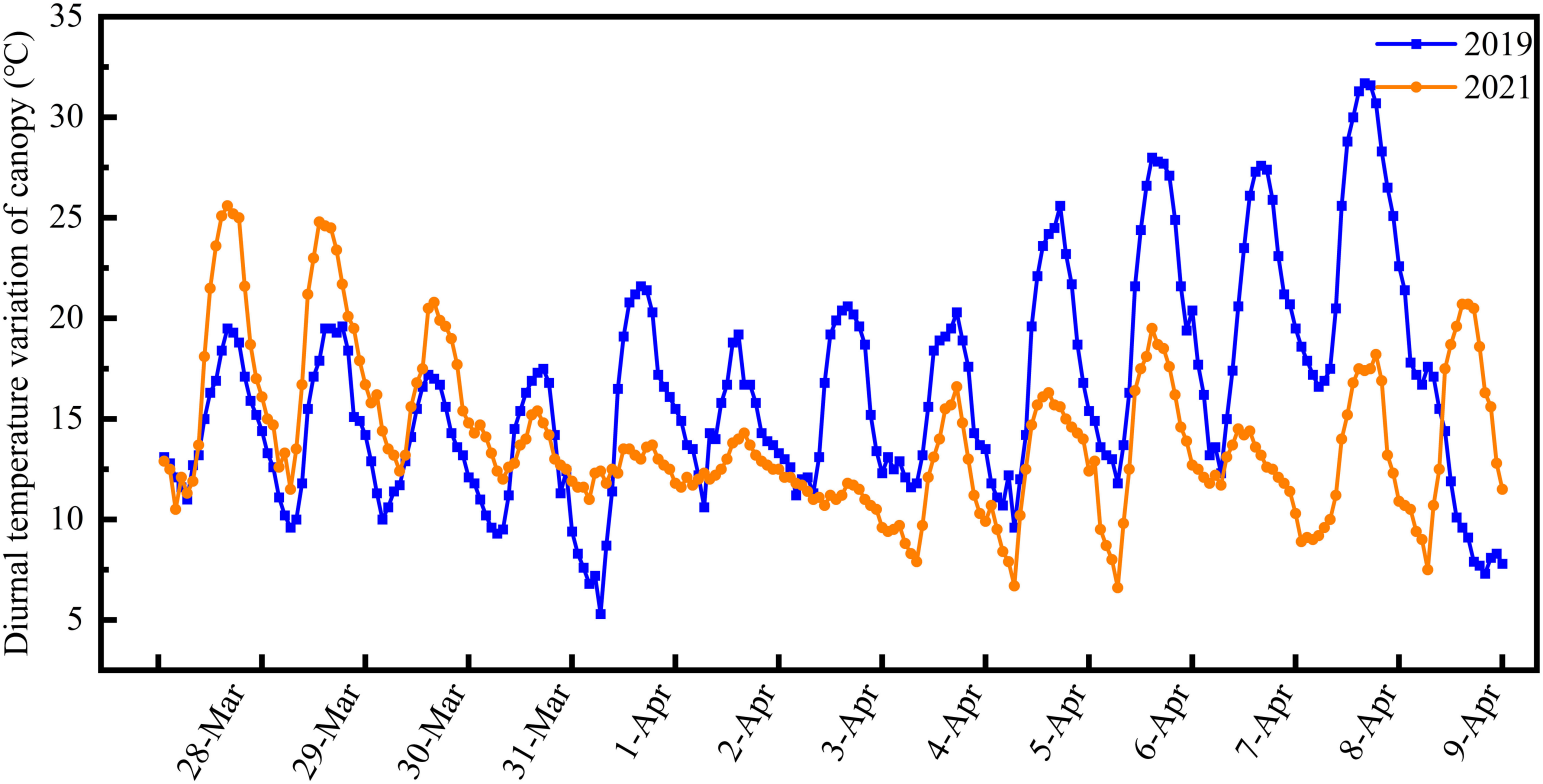
Diurnal variation in canopy temperature in the control group during the two-year field study. The wheat reached another separation stage on 3 April 2019 and 28 March 2021. Canopy temperature was recorded at 80-minute intervals.

The differentiation process of the young wheat ears was observed using a microscope (SZX16, Olympus, Japan). After differentiation of the young ears to the anther separation stage, 90 pots per variety were moved from the experimental field and placed in an artificial climate chamber for LTS treatment at booting. LTS treatment was carried out for 24 h on 3 April 2019 and 28 March 2021, respectively, as follows: at 2°C, 0°C or −2°C from 19:00 to 07:00 followed by 5°C from 07:00 to 19:00. Humidity was maintained at 70%. Pots were then returned to the field and reburied. Pots of each variety that remained in the field and did not undergo LTS treatment at booting were used as a control. Diurnal changes in the wheat canopy temperature of the field control group in 2019 and 2021 are shown in **Figure 1**.

### 2.3 Measurements

#### 2.3.1 Photosynthetic parameters of the flag leaves

For analysis of photosynthetic parameters in the flag leaves, 10 pots showing consistent growth and development were randomly selected from each treatment between 09:00 to 11:00 in the morning on a clear and cloudless day. Samples were obtained at the heading stage, anthesis stage and filling stage then *P*_n_, *G*_s_ and *T*_r_ of the flag leaves were measured using a Photosynthesis-Fluorescence system (Li-6400XT, Li-Cor Inc, USA) with a 2×3 cm standard leaf chamber. Leaf chamber parameters were set as follows: an ambient atmospheric CO_2_ concentration (*C*_a_) of 400 μ·mol^−1^, light intensity of 1200 μmol·m^−2^·s^−1^, temperature of 20°C, and vapor pressure deficit (VPD) of 1.5 kPa.

#### 2.3.2 Starch granules morphology

Wheat ears showing anthesis on the same date, and consistent growth and development were marked. At maturity, 5 to 10 of the marked ears showing consistent growth were then sampled. From these, six to seven grains from the middle of the ear and one to two grains from the base of the spikelet were harvested then dissected down the central axis with a razor blade to obtain a cross section of the grain endosperm. The samples were then fixed on a copper column using double-sided carbon glue and gold-plated using an ion sputtering device. The ultrastructure of the endosperm starch granules was then observed using a scanning electron microscope (S-4800, Hitachi, Japan).

#### 2.3.3 Isotopic ^13^C abundance

For each treatment, 10 single wheat stems showing consistent growth were selected then the upper three leaves on the main stem of single plants were marked between 10:00–11:00 am on the same day as LTS treatment. Before ^13^CO_2_ labeling, the marked unfolded leaves were sealed in a polyvinyl chloride transparent plastic film bag, which was then injected with 5 mL of ^13^CO_2_ using a medical injector. The sealed bag was removed after photosynthetic assimilation under natural light for 60 min. The marked flag leaves were then sampled three days later, while the grains from the marked plants were sampled at maturity. The samples were ground using an analytical grinder (A11 basic, IKA, Germany) after de-enzyming and drying then passed through a 100-mesh sieve. An elemental analysis-stable isotope ratio mass spectrometer (EA-IRMS) (Integra 2, Sercon, UK) was then used to determine the abundance of ^13^C in 50 mg leaf and grain samples, with three repetitions per measurement. ^13^C abundance was then calculated as follows:


(1) $$\delta^{13}\text{C abundance (‰)=}\left[\frac{\text{R}\left({\text{C}}^{13}/{{\text{C}}^{12}}_{\text{sample}}\right)}{\text{R}\left({\text{C}}^{13}/{{\text{C}}^{12}}_{\text{VPDB}}\right)}-1\right]\text{x}1000$$

where *R* (^13^C/^12^C_VPDB_) is the carbon isotope abundance ratio of the international standard Vienna Pee Dee Belemnite (VPDB). The analytical precision of ^13^C abundance was set at ± 0.2‰.

#### 2.3.4 Dry matter accumulation and distribution

After anthesis, wheat ears that bloomed on the same day were marked. At anthesis and maturity, 30 ears showing consistent growth were then selected from each plot. At anthesis, the samples were divided into the stem sheath, leaf and ear, while at maturity, they were divided into the stem sheath, leaf, glume of the leaf rachis, and grain. The samples were fixed at 105°C for 30 min then dried at 75°C to a constant weight. The dry matter distribution of each organ was then weighed, and dry matter accumulation was calculated as follows:


(2) $$\begin{array}{c} \mathrm{Translocation}\;\mathrm{amount}\;\mathrm{of}\;\mathrm{pre}-\mathrm{anthesis}\;\mathrm{stored}\;\mathrm{dry}\;\mathrm{matterfrom}\;\mathrm{vegetative}\;\mathrm{organs}\;\mathrm{to}\;\mathrm{grains}\\ \left(g\cdot \mathrm{po}t^{-1}\right)\;=\;\mathrm{amount}\;\mathrm{of}\;\mathrm{dry}\;\mathrm{matter}\;\mathrm{in}\;\mathrm{the}\;\mathrm{vegetative}\;\mathrm{organs}\;\mathrm{at}\;\mathrm{anthesis}\;-\;\mathrm{amount}\;\mathrm{of}\;\mathrm{dry}\;\mathrm{matter}\\ \mathrm{in}\;\mathrm{the}\;\mathrm{vegetative}\;\mathrm{organs}\;\mathrm{at}\;\mathrm{maturity} \end{array}$$

(3) $$\begin{array}{c} \mathrm{Contribution}\;\mathrm{rate}\;\mathrm{of}\;\mathrm{pre}-\mathrm{anthesis}\;\mathrm{stored}\;\mathrm{dry}\;\mathrm{matter}\;\mathrm{to}\;\mathrm{grains}\;\left(\%\right)\;=\mathrm{translocation}\;\mathrm{amount}\;\mathrm{of}\\ \mathrm{pre}-\mathrm{anthesis}\;\mathrm{stored}\;\mathrm{dry}\;\mathrm{matte}\;\mathrm{from}\;\mathrm{vegetative}\;\mathrm{organs}\;\mathrm{to}\;\mathrm{grains}\;/\;\mathrm{amount}\;\mathrm{of}\;\mathrm{dry}\;\mathrm{matter}\;\mathrm{in}\;\mathrm{the}\\ \mathrm{grains}\;\mathrm{at}\;\mathrm{maturity}\;\times \;100 \end{array}$$

(4) $$\begin{array}{c} \mathrm{Amount}\;\mathrm{of}\;\mathrm{post}-\mathrm{anthesis}\;\mathrm{transfer}\;\mathrm{of}\;\mathrm{accumulated}\;\mathrm{dry}\;\mathrm{matterinto}\;\mathrm{grains}\;\left(g\cdot \mathrm{po}t^{-1}\right)\;=\;\mathrm{amount}\;\mathrm{of}\\ \mathrm{dry}\;\mathrm{matter}\;\mathrm{in}\;\mathrm{thegrainsat}\;\mathrm{maturity}\;-\;\mathrm{translocation}\;\mathrm{amount}\;\mathrm{of}\;\mathrm{pre}-\mathrm{anthesis}\;\mathrm{stored}\;\mathrm{dry}\;\mathrm{matte}\\ \mathrm{from}\;\mathrm{vegetative}\;\mathrm{organs}\;\mathrm{to}\;\mathrm{grains} \end{array}$$

(5) $$\begin{array}{c} \mathrm{Contribution}\;\mathrm{rate}\;\mathrm{of}\;\mathrm{post}-\mathrm{anthesis}\;\mathrm{accumulated}\;\mathrm{dry}\;\mathrm{matter}\;\mathrm{to}\;\mathrm{grains}\;\left(\%\right)\;=\;\mathrm{amount}\;\mathrm{of}\;\mathrm{post}-\\ \mathrm{anthesis}\;\mathrm{transfer}\;\mathrm{of}\;\mathrm{dry}\;\mathrm{matterinto}\;\mathrm{grains}\;/\;\mathrm{amount}\;\mathrm{of}\;\mathrm{accumulated}\;\mathrm{dry}\;\mathrm{matter}\;\mathrm{in}\;\mathrm{the}\;\mathrm{grains}\;\mathrm{at}\\ \mathrm{maturity}\;\times \;100 \end{array}$$

From 10 days after anthesis to maturity, 15–20 wheat ears showing uniform growth were selected every five days then the grains were removed. The seeds were fixed at 105°C for 30 min then dried at 75°C to a constant weight, weighed and converted into the 1000-grain weight. The logistic equation *Y* = K/(1+e^(a+b^*^t^*^)^) was used to associate the variation in grain weight (*Y*) with the number of days after anthesis (*t*), where K is the fitted maximum grain weight, and a and b are parameters (Darroch and Baker, 1990). The first and second derivations of the equation were then used to derive the following:

Duration of the incremental filling period (*T*_1_):


(6) $$T_{1}=\frac{a-1.317}{b}$$

Duration of the rapid filling period (*T*_2_):


(7) $$T_{2}=\frac{a+1.317}{b}-\frac{a-1.317}{b}$$

Duration of the slow filling period (*T*_3_):


(8) $$T_{3}=T-T_{1}-T_{2}$$

Appearance time of maximum grain filling (*T*_max_):


(9) $$T_{max}=-\frac{a}{b}$$

Number of filling days (*T*):


(10) $$T=\frac{\ln\frac{1}{9}-a}{b}$$

Mean filling rate (*R*):


(11) $$R=\frac{K}{T}$$

Maximum filling rate (*R*_max_):


(12) $$R_{max}=-\frac{Kb}{4}$$

#### 2.3.5 Activities of Starch Phosphorylase (Pho1) and Disproportionating Enzyme (Dpe1)

Fresh grain samples (1 g) were obtained from 10 to 20 d after anthesis then ground into pulp in a freezing grinder. Next, 1 ml of 80% methanol was added then the samples were incubated overnight at −20°C. They were then centrifuged at 8000 ×g at 4°C for 15 min. The supernatant was then passed through a C-18 solid phase extraction column, and dried in vacuum. PBS buffer (pH7.4) was added before loading to a final volume of 1 ml. After mixing, the samples were placed at room temperature for 30 min. They were then centrifuged at 10,000 ×g for 10 min at 4°C before storing at 4°C until use.

The activity of Pho1activity was determined according to the method of Hwang et al. (2010). Briefly, the crude enzyme solution was mixed with 100 mmolL^−1^ Mes–NaOH (pH6.5) and 20 mmol L^−1^ Glc-1-P to prepare the reaction solution. Two-parts solution A (12% w/v L- ascorbic acid in 1 mol·L^−1^ HCl) and one-part solution B (2% w/v ammonium molybdate tetrahydrate in deionized water) were then mixed to make solution C. Next, 0.2 mL of solution C was added to the reaction solution to stop the enzyme reaction. After 5 min at room temperature, 0.2 ml of solution F (2% w/v sodium citrate trihydrate and 2% v/v acetic acid in deionized water solution) was added to stop color development. The absorbance at 650 nm was then determined.

The activity of Dpe1 enzyme was determined according to the method of Akdogan et al. (2011) with slight modifications. Briefly, 50 μL of crude enzyme extract was mixed with 50 μL of maltotriose then placed in a water bath at 30°C for 30 min. The reaction was terminated by placing the sample in a boiling water bath for 10 min. The activity of Dpe1 was calculated by measuring the release of Glc.

#### 2.3.6 Quantitative assays of *AGPase*, *GBSSI*, *SSSI*, *SSSII* and *Pho1* expression

Total RNA was extracted from each sample using a RNAprep Pure Plant Plus Kit (Tiangen Biotech, China). cDNA was then synthesized using 12 μL samples of the obtained RNA. HiScript IIQ RT SuperMix was used for qPCR (+gDNA wiper) (Vazyme Biotechnology, China). The synthesized cDNA was then detected using a real-time quantitative pcr detecting system (Gentier 96E, Tianlong technology, China). qRT-PCR was selected using a Hieff UNICON^®^ Universal Blue qPCR SYBR Green Master Mix test kit (Yeasen, China), with three technical repeats per sample. The data were analyzed using the 2^−ΔΔCt^ method. The wheat *ACTIN* gene was selected as a reference gene, and the detection primers were F_W_:5’-CTCCTCTCTGCGCCAATCGT and R_ev_:5’-TCAGCCGAGCGGGAAATTGT. See **Table 2** for details of the gene and detection primers (Sangon Biotechnology, China).

**Table 2:** qRT-PCR was used to detect the primer information corresponding to the gene.

Gene name	NCBI accession number	qRT-PCR primer	bp
*AGPase*	Z21969	F: GCGAACTCAAGAACGCGATG	114
		R: TCTTTGTGTTCTCCCCGACG	
*GBSSI*	AF286320	F: CGTCTCCGAGATCAAGGTCG	183
		R: AAGCGTAGCTGGTTGTCCTC	
*SSSI*	AF091803	F: TGCTCGAAGGGATTGCTGAG	97
		R: GCTTGAGGTTGCTCATTCGC	
*SSSII*	AF155217	F: CTCCCAGGCTGGACATTGAC	215
		R: TTCAAAGGAGCCCGCATCAT	
*Pho1*	EU595762	F: ACGGGGAAGTTGCTTGTTCA	109
		R: CGCCCTTTTTCTCTGCTGTC	

#### 2.3.7 Starch content

From 10 to 35 days after anthesis, 15–20 wheat ears from each treatment were selected and threshed every 5 days. The seeds were fixed at 105°C for 30 min then dried at 75°C to a constant weight. They were then ground using an analytical grinder (IKA A11 basic), passed through a 100-mesh sieve then weighted into 0.1g samples. The precipitate remaining after the extraction of total soluble sugars was dried at 60°C then shaken with 2 ml of distilled water. The solution was then boiled for 20 min, cooled before adding 2 mL of 9.2 mol·L^−1^ HClO_4_. The samples were then shaken for 10 min, mixed with 6 mL of distilled water then centrifuged at 5,000 rpm·min^-1^ for 15 min. The supernatant samples were then decanted into a 50 mL volumetric flask. The process was repeated three times to a constant volume each time. A 0.1 mL sample of extract was then added to 4 mL 0.2% anthrone, and boiled for 15 min. The OD at 620 nm was then measured after cooling.

#### 2.3.8 Number of grains per spike and the 1000-grain weight

After maturity, ears were sampled from 20 random pots previously unsampled. The number of grains per spike and the 1000-grain weight were then determined.

### 2.4 Data analysis

Excel 2019 was used for data sorting, and statistical significance was determined using SPSS 26.0 software. Duncan’s method was used for multiple comparisons between treatments, and Origin 2017 was used to generate graphs.

## 3 Results

### 3.1 LTS decreased photosynthetic parameters of the flag leaves

LTS at booting significantly reduced the *P*_n_ of the flag leaves at heading, anthesis and grain filling, as well as the *G*_s_ and *T*_r_ at grain filling (*P*< 0.05, **Table 3**). The *P*_n_, *G*_s_ and *T*_r_ of the flag leaves of both varieties decreased at each growth stage with decreasing temperature, and were lowest at −2°C. At the grain filling stage, *P*_n_, *G*_s_ and *T*_r_ decreased by 52.13%, 53.85% and 55.85% in low-temperature slow variety Yannong 19, compared with the control, respectively, after LTS treatment at −2°C.

**Table 3:** Effect of LTS at booting on photosynthetic parameters of wheat flag leaf.

Variety	Treatment	Heading Stage			Anthesis stage			Filling Stage		
		Net photosynthetic rate µmol CO_2_ m^−2^ s^−1^	Stomatal conductance mol H_2_O m^−2^ s^−1^	Transpiration rate mmol H_2_O m^−2^ s^−1^	Net photosynthetic rate µmol CO_2_ m^−2^ s^−1^	Stomatal conductance mol H_2_O m^−2^ s^−1^	Transpiration rate mmol H_2_O m^−2^ s^−1^	Net photosynthetic rate µmol CO_2_ m^−2^ s^−1^	Stomatal conductance mol H_2_O m^−2^ s^−1^	Transpiration rate mmol H_2_O m^−2^ s^−1^
Yannong 19	control	11.49a ± 0.09	0.09a ± 0.00	2.13a ± 0.04	18.60a ± 0.44	0.16a ± 0.00	4.06a ± 0.00	16.67a ± 0.06	0.26a ± 0.00	7.09a ± 0.05
	2°C	10.38b ± 0.02	0.08b ± 0.00	2.08a ± 0.00	15.26b ± 0.99	0.16a ± 0.00	4.04a ± 0.01	14.09b ± 0.05	0.17b ± 0.00	4.90b ± 0.02
	0°C	5.51c ± 0.14	0.03c ± 0.00	0.85b ± 0.00	12.70c ± 0.09	0.16a ± 0.00	4.02a ± 0.00	11.56c ± 0.10	0.13c ± 0.00	4.16c ± 0.00
	−2°C	3.60d ± 0.05	0.02d ± 0.00	0.70c ± 0.00	8.91d ± 0.02	0.07b ± 0.00	2.14c ± 0.01	7.98d ± 0.12	0.12d ± 0.00	3.13d ± 0.05
Wanmai 52	control	12.70a ± 0.09	0.16a ± 0.00	4.06a ± 0.00	18.50a ± 0.01	0.32a ± 0.00	6.36a ± 0.01	16.39a ± 0.03	0.17a ± 0.00	7.29a ± 0.01
	2°C	10.07b ± 0.01	0.06b ± 0.00	1.51b ± 0.00	13.66b ± 0.44	0.18b ± 0.01	3.91b ± 0.19	12.33b ± 0.08	0.13b ± 0.00	3.15b ± 0.04
	0°C	7.61c ± 0.08	0.04c ± 0.00	1.18c ± 0.03	10.87c ± 0.06	0.11c ± 0.00	2.97c ± 0.00	7.20c ± 0.05	0.12b ± 0.00	3.14b ± 0.00
	−2°C	5.64d ± 0.04	0.04c ± 0.00	1.14c ± 0.03	8.07d ± 0.10	0.11c ± 0.00	2.65d ± 0.08	5.86d ± 0.01	0.05c ± 0.00	1.81c ± 0.01

Control: wheat that was not exposed to low-temperature stress (LTS) treatment at booting and remained in the field; 2, 0 and −2°C: wheat placed in an artificial climate chamber at booting and treated for 24 h at 2, 0 or −2°C from 19:00 until 07:00 then at 5°C from 07:00 to 19:00. Different lowercase letters indicate a significant difference between treatments according to Duncan’s multiple range test (P< 0.05). The number after “±”indicates the standard error.

### 3.2 LTS hindered the development of starch granules in endosperm

**Figure 2:**
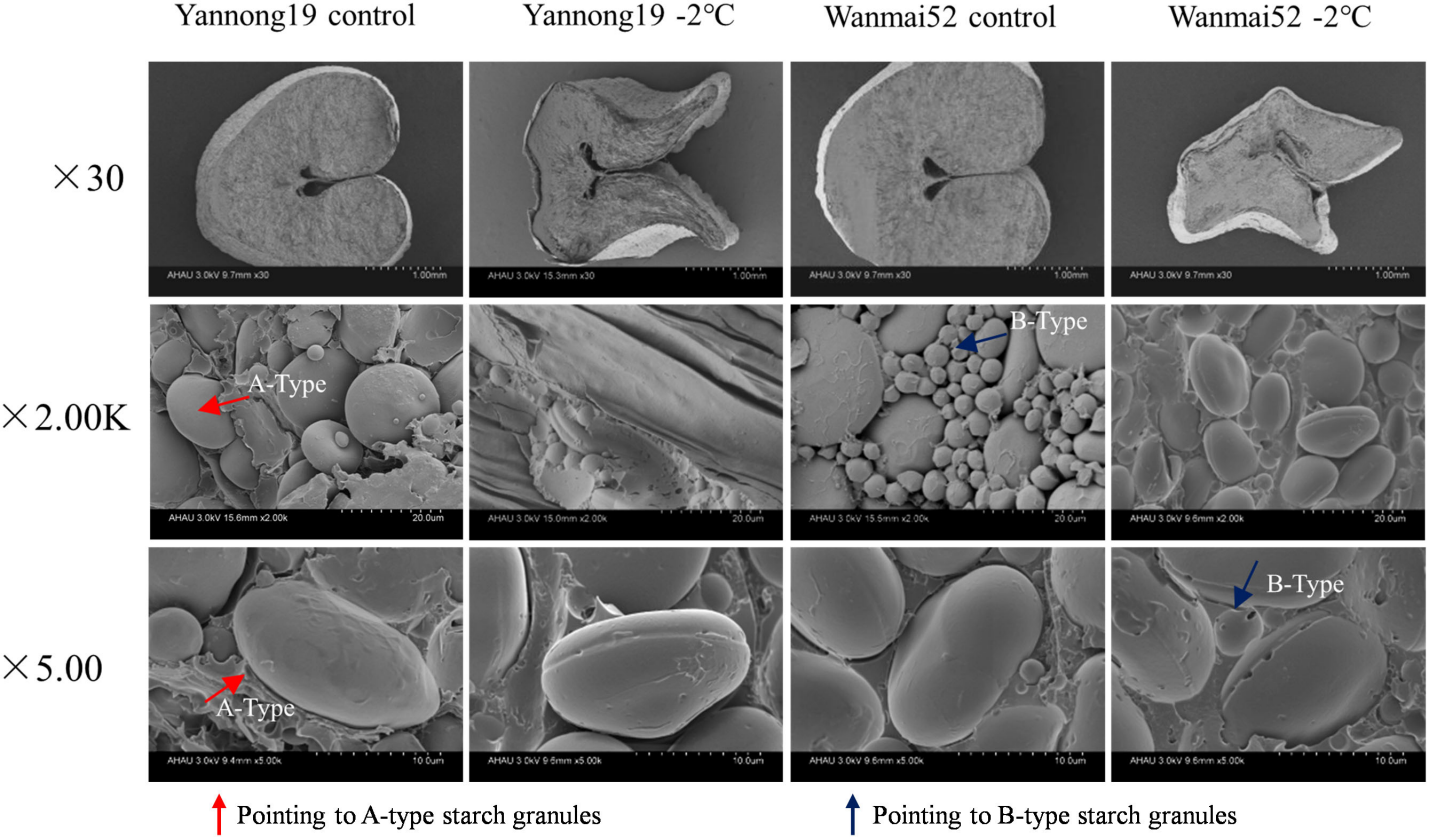
Effect of LTS at booting on the starch grain morphology of mature wheat grains. Morphological changes were observed under a scanning electron microscope at × 30, × 2.00K, and ×5.00K. A-Type: a-type starch granules; B-Type: b-type starch granules. Control: wheat that was not exposed to LTS treatment at booting and remained in the field; −2°C: wheat placed in an artificial climate chamber at booting and treated for 24 h at −2°C from 19:00 until 07:00 then at 5°C from 07:00 to 19:00.

LTS at booting affected the development of starch granules in the endosperm (**Figure 2**). In control treatment, the cross-section of the wheat endosperm was plump, while under −2°C LTS, the cross-section was shrunken, and the volume reduced. The plumpness of both A- and B-type starch granules decreased, and obvious equatorial grooves were observed on the surface of the A-type granules. Meanwhile, the number of B-type starch granules decreased. LTS treatment at −2°C also resulted in the appearance of micropores in the equatorial grooves of the A-type starch granules in Wanmai 52, but not Yannong 19.

### 3.3 LTS reduced ^13^C abundance

**Figure 3:**
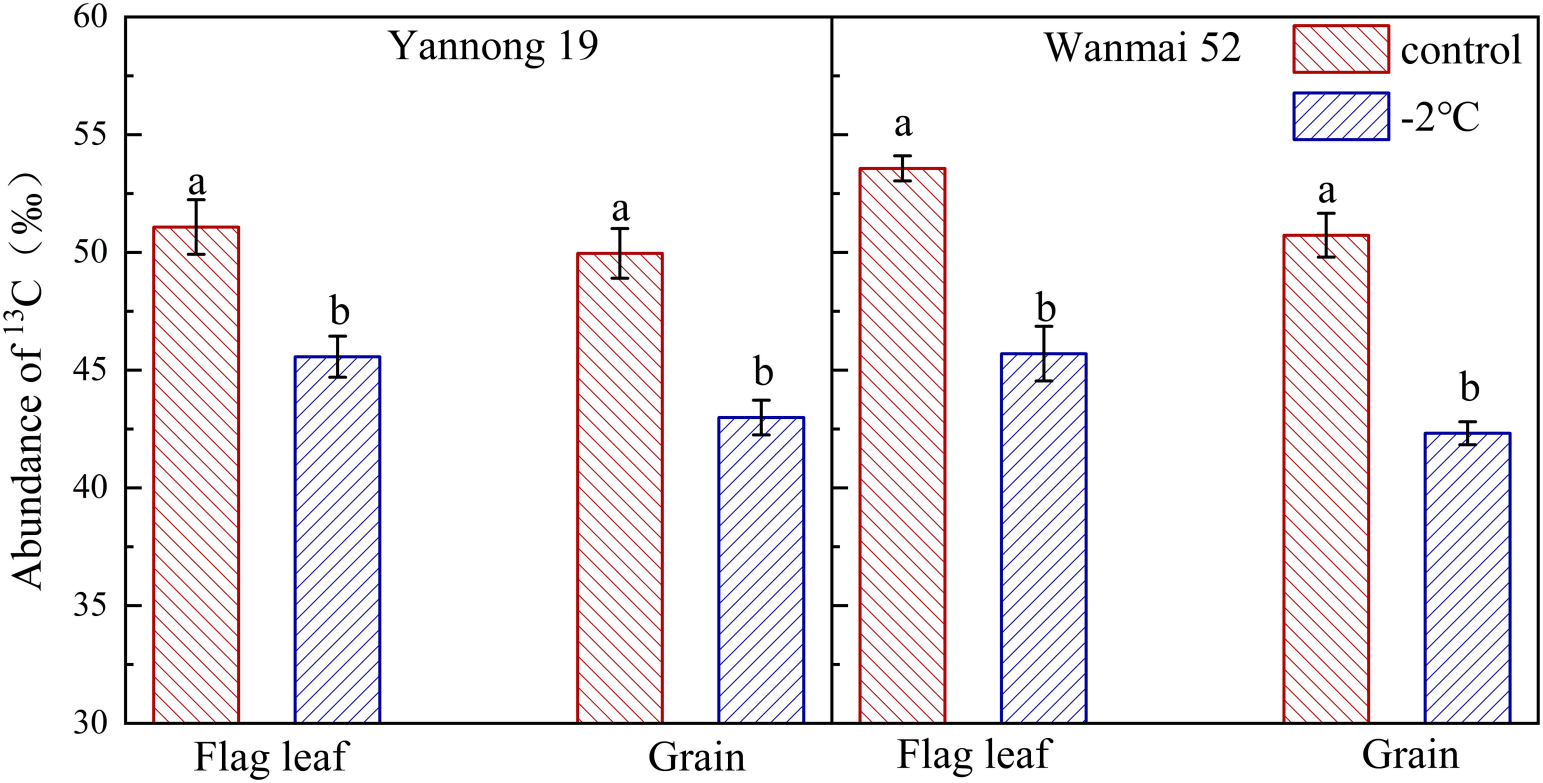
Effect of LTS at booting on the abundance of ^13^C in the flag leaves and grains. Control and −2°C treatment are as in **Figure 2**. Flag leaves were labeled with ^13^CO_2_ then sampled three days later, while grains were sampled at maturity. Different lowercase letters indicate a significant difference between treatments according to Duncan’s multiple range test (*P<* 0.05).

LTS at booting also affected the distribution of photosynthetic carbon products, with a significant decrease in the abundance of ^13^C in the flag leaves and wheat grains at maturity according to ^13^CO_2_ labeling (*P<* 0.05, **Figure 3**). Compared with the control, the abundance of ^13^C in the flag leaves decreased by 10.79% in Yannong 19 and 14.69% in Wanmai 52 after LTS treatment at −2°C, while the abundance of ^13^C in the grains decreased by 13.95% and 16.58%, respectively. LTS treatment at booting caused a greater decrease in ^13^C abundance in the grains of Wanmai 52 compared to Yannong 19.

### 3.4 LTS reduced dry matter accumulation and distribution to the grains

**Figure 4:**
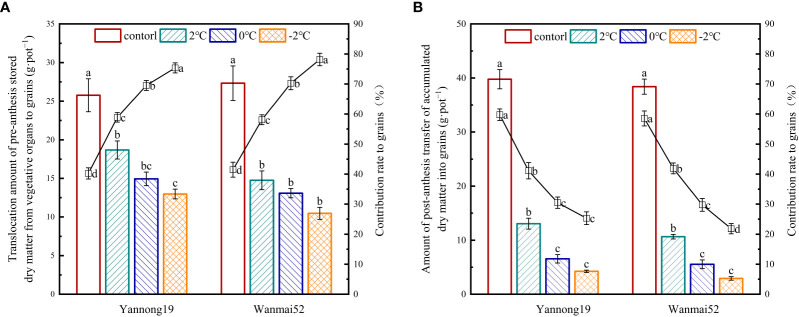
Translocation amount of pre-anthesis stored dry matter from vegetative organs to grains **(A)** and amount of post-anthesis transfer of accumulated dry matter into grains **(B)**, and the contribution rate to the grains following LTS at booting. The histogram shows translocation amount of pre-anthesis stored dry matter from vegetative organs to grains **(A)** and amount of post-anthesis transfer of accumulated dry matter into grains **(B)**. The line chart shows the contribution rates of the above two indexes to mature grains. Control: wheat that was not exposed to LTS treatment at booting and remained in the field; 2, 0 and −2°C: wheat placed in an artificial climate chamber at booting and treated for 24 h at 2, 0 or −2°C from 19:00 until 07:00 then at 5°C from 07:00 to 19:00. Different lowercase letters indicate a significant difference between treatments according to Duncan’s multiple range test (*P<* 0.05).

LTS at booting caused a significant reduction in translocation amount of pre-anthesis stored dry matte from vegetative organs to grains and amount of post-anthesis transfer of accumulated dry matte into grains (*P<* 0.05, **Figure 4**). Moreover, with decreasing temperature, the greater the decrease in the contribution rate of post-anthesis accumulated dry matter to grains decreased, with lowest values observed at −2°C. Compared with the control, amount of post-anthesis transfer of accumulated dry matte into grains decreased by 72.25%, 85.57%, and 92.39% in low-temperature sensitive variety Wanmai 52, while the contribution rate of post-anthesis accumulated dry matter to grains decreased by 28.39%, 49.17%, and 62.71%, respectively, following LTS treatment at 2°C, 0°C, and −2°C.

LTS at booting also caused a significant reduction in the distribution rate of dry matter in the grains at maturity (*P<* 0.05, **Table 4**), while the distribution of dry matter in the stem, sheath and leaf at maturity increased significantly (*P<* 0.05). Moreover, the distribution rate of dry matter in the grains at maturity decreased continuously with decreasing temperature at booting.

**Table 4:** Effect of LTS at booting on the distribution ratio of dry matter in different organs of wheat.

Variety	Treatment	Anthesis stage			Maturity stage			
		Stem-sheath (%)	Leaf (%)	Spike (%)	Stem-sheath (%)	Leaf (%)	Spike-stalk+glume (%)	Grain (%)
Yannong 19	control	61.78a ± 0.25	18.45c ± 0.28	19.77a ± 0.33	31.59b ± 0.29	8.44c ± 0.74	14.21b ± 0.45	45.76a ± 1.19
	2°C	60.65ab ± 1.17	20.19bc ± 0.99	19.16ab ± 0.80	36.47a ± 0.57	9.99b ± 0.24	15.01b ± 0.38	38.52b ± 0.84
	0°C	60.12ab ± 0.63	21.40b ± 0.60	18.48ab ± 0.33	35.02a ± 0.95	11.89a ± 0.24	17.67a ± 0.75	35.42c ± 0.33
	−2°C	58.37b ± 0.57	23.65a ± 0.49	17.98b ± 0.21	36.82a ± 0.19	13.19a ± 0.27	17.80a ± 0.11	32.19d ± 0.39
Wanmai 52	control	66.46a ± 0.19	16.69c ± 0.33	16.85a ± 0.18	32.35d ± 0.24	9.67b ± 0.21	14.37a ± 0.45	43.61a ± 0.71
	2°C	65.32ab ± 0.79	18.37bc ± 0.70	16.31ab ± 0.51	37.63c ± 0.51	11.65a ± 0.27	15.25a ± 0.45	35.47b ± 0.28
	0°C	64.52b ± 0.56	19.40ab ± 0.88	16.08ab ± 0.47	40.50b ± 0.34	11.56a ± 0.20	15.50a ± 0.41	32.44c ± 0.28
	−2°C	63.91b ± 0.31	20.49a ± 0.27	15.60b ± 0.18	42.87a ± 0.41	11.15a ± 0.30	15.47a ± 0.29	30.51d ± 0.23

Control and 2, 0 and −2°C treatments are as in **Table 3**. Different lowercase letters indicate a significant difference between treatments according to Duncan’s multiple range test (P< 0.05). The number after “±”indicates the standard error.

### 3.5 Grain filling parameters changed after LTS

A logistic equation was used to fit the grain-filling dynamics of wheat under LTS treatment (**Table 5**). LTS at booting reduced the maximum theoretical 1000-grain weight and, compared with the control, caused a decrease in the duration of rapid (*T*_2_) and slow filling (*T*_3_). The effective number of filling days also decreased (*T*), while the appearance time of the maximum grain filling rate was advanced (*T*_max_). LTS at booting also caused a reduction in the mean (*R*) and maximum filling rate (*R*_max_) compared with the control, and the lower the temperature, the smaller the value.

**Table 5:** Summary of the dry matter accumulation model and the parameters of wheat grain after LTS treatment during the booting.

Variety	Treatment	Model	Decision coefficient (*R*^2^)	*T*_1_ (d)	*T*_2_ (d)	*T*_3_ (d)	*T* (d)	*T*_max_ (d)	*R* (g·1000 grain^−1^·d^−1^)	*R*_max_ (g·1000 grain^−1^·d^−1^)
Yannong 19	control	*Y*=47.5044/(1+e^(3.1679−0.172972^*^t^*^)^)	0.9961^**^	10.6988	15.2254	5.0880	31.0123	18.3116	1.3573	2.0546
	2°C	*Y*=41.1449/(1+e^(3.1713−0.176569^*^t^*^)^)	0.9974^**^	10.5000	14.9151	4.9843	30.3993	17.9575	1.1756	1.8165
	0°C	*Y*=37.0516/(1+e^(3.2136−0.184370^*^t^*^)^)	0.9976^**^	10.2852	14.2842	4.7735	29.3429	17.4273	1.0586	1.7081
	−2°C	*Y*=33.8635/(1+e^(3.4276−0.193964^*^t^*^)^)	0.9955^**^	10.8794	13.5773	4.5372	28.9939	17.6680	0.9675	1.6424
Wanmai 52	control	*Y*=47.6889/(1+e^(3.7161−0.198575^*^t^*^)^)	0.9947^**^	12.0800	13.2628	4.4321	29.7750	18.7115	1.3625	2.3678
	2°C	*Y*=40.6325/(1+e^(3.7941−0.204816^*^t^*^)^)	0.9933^**^	12.0952	12.8613	4.2980	29.2545	18.5259	1.1609	2.0804
	0°C	*Y*=35.0907/(1+e^(3.9810−0.221257^*^t^*^)^)	0.9978^**^	12.0380	11.9024	3.9775	27.9179	17.9892	1.0026	1.9414
	−2°C	*Y*=32.7670/(1+e^(4.0707−0.221920^*^t^*^)^)	0.9970^**^	12.4096	11.8702	3.9668	28.2466	18.3447	0.9362	1.8177

Control and 2, 0 and −2°C treatments are as in **Table 3**. Y: the weight of 1000 grains; t: the day after anthesis; T_1_, T_2_, and T_3_: the duration of grain filling increasing period, the fast increasing period, and slow increasing period; T_max_: the time of maximum grain filling rate; T: the duration of grain filling; R: average grain filling rate; R_max_: maximum grain filling rate. ^**^indicate significant differences at the 0.01 level.

### 3.6 The activities of Pho1 and Dpe1 decreased after LTS

**Figure 5:**
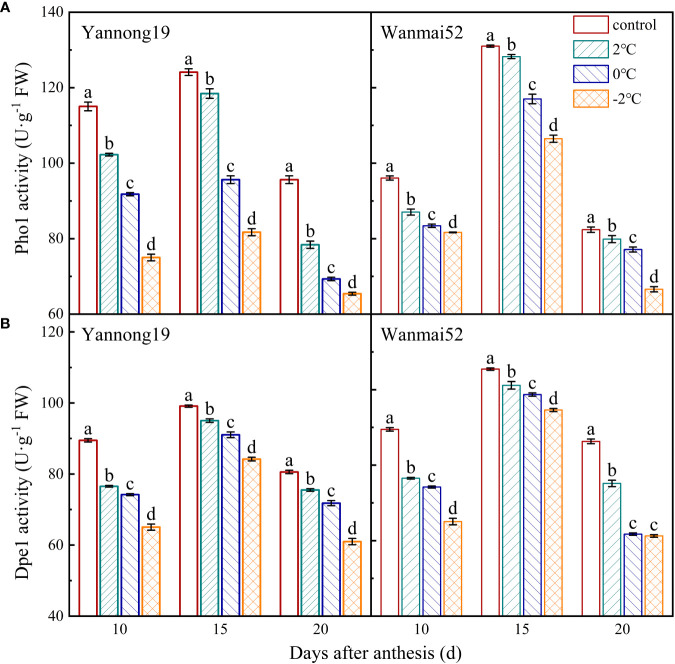
Effects of LTS at booting on the activities of Pho1 **(A)** and Dpe1 **(B)** in the wheat grains. Control, and 2, 0 and −2°C treatment are as in **Figure 4**. Different lowercase letters indicate a significant difference between treatments according to Duncan’s multiple range test (*P<* 0.05).

The activities of Pho1 **(** **Figure 5A**) and Dpe1 (**Figure 5B**) first increased and then decreased within 10–20 d after anthesis, reaching a maximum at about 15 d after anthesis. Compared with the control, the activities of Pho1 and Dpe1 in the treated decreased significantly with decreasing temperature (*P*< 0.05). Taking the 15 d after anthesis of Yannong 19 as an example, treatment at 2°C, 0°C and −2°C resulted in a reduction in the Pho1 activity of 4.57%, 22.96%, and 34.17%, respectively, and the Dpe1 enzyme activity decreased by 4.12%, 8.14%, and 15.04%, respectively.

### 3.7 LTS reduced expression of genes related to starch synthesis

**Figure 6:**
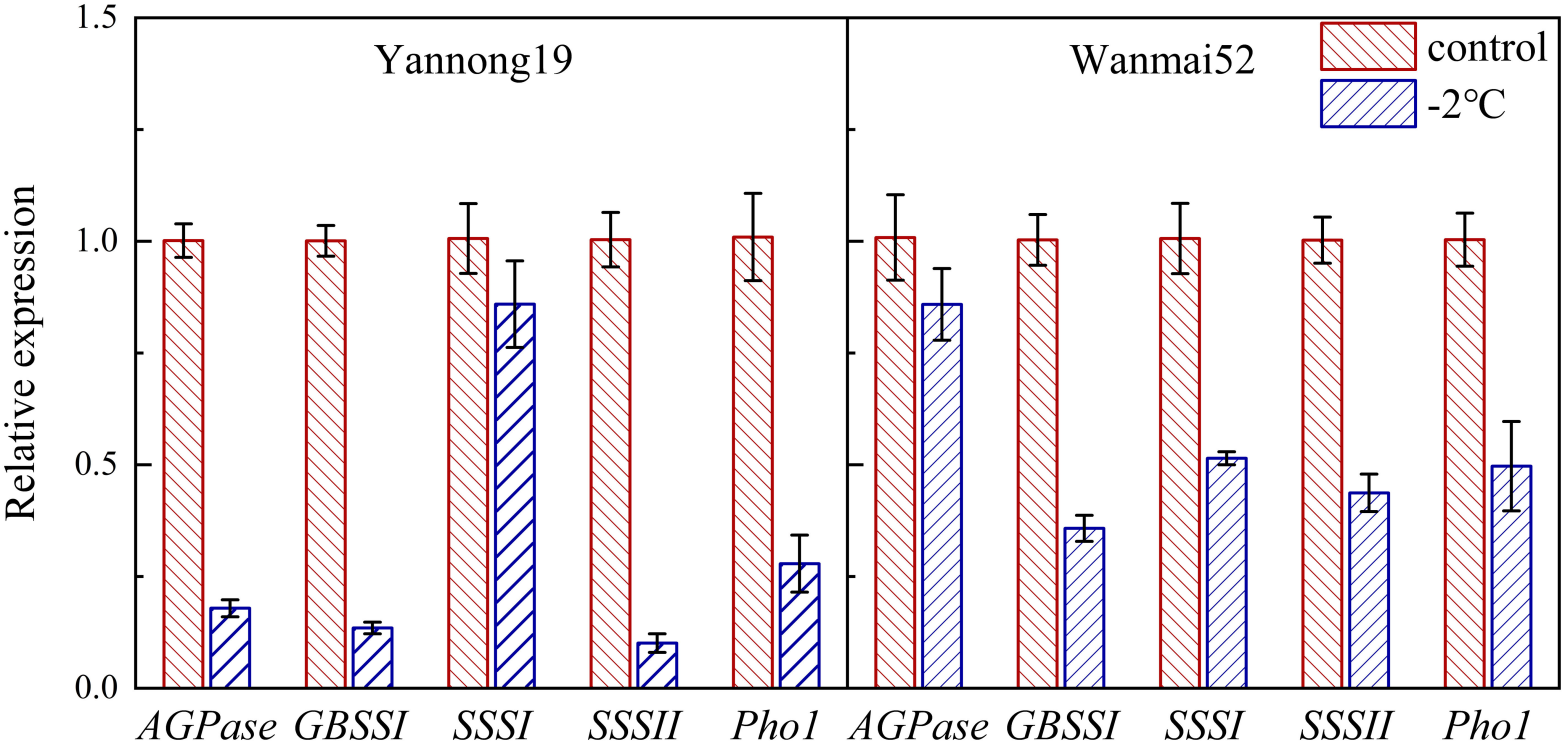
Effect of LTS at booting on the relative expression of starch synthase genes (*AGPase, GBSSI, SSSI, SSSII*, and *Pho1*) in the wheat grains. Control and −2°C treatment are as in **Figure 2**.

q-PCR was used to determine the effect of LTS on gene expression of enzymes related to starch synthesis in wheat grains 20 d after anthesis (**Figure 6**). LTS treatment at −2°C resulted in down-regulation of *AGPase, GBSSI, SSSI, SSSII* and *Pho1* expression in both wheat varieties. Compared with the control, Compared with the control, the relative expression levels of these genes in sensitive variety Wanmai 52 decreased by 14.84%, 64.32%, 48.87%, 56.41% and 50.46%, respectively.

### 3.8 LTS decreased starch content of the grains

**Figure 7:**
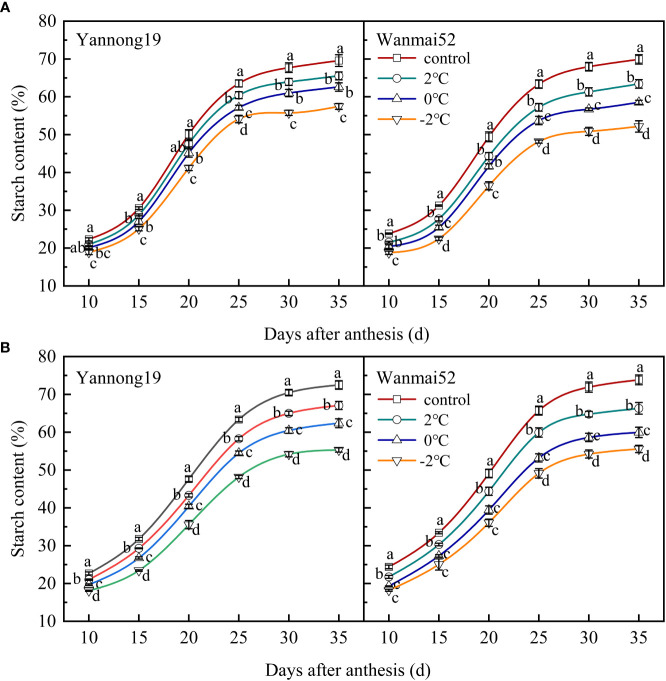
Effect of LTS at booting on the starch content of the grains in 2018–2019 **(A)** and 2020–2021 **(B)**. Control and 2, 0 and −2°C treatment are as in **Figure 4**. Different lowercase letters indicate a significant difference between treatments according to Duncan’s multiple range test (*P<* 0.05).

During the grain filling process, a gradual increase in the starch content was observed with time, in accordance with the logistic regression model. A rapid increase was observed from 15 to 25 d after anthesis followed by a more gradual increase thereafter then a gradual plateau (**Figure 7**). LTS at booting caused a significant decrease in the starch content of the wheat grains 25 d after anthesis (*P<* 0.05), and the lower the temperature, the greater the decrease. At 35 d after anthesis, the starch content of the grains reached a significant level between treatments in Wanmai 52. Take the Wanmai 52 in 2021 year results as an example, compared with the control, the starch content of the grains decreased by 10.20%, 18.80%, and 24.74% at 35 d after anthesis, respectively, following LTS treatment at 2°C, 0°C, and −2°C.

### 3.9 LTS decreased the number of grains per spike and the 1000-grain weight

LTS at booting resulted in a significant reduction in the number of grains per spike and the 1000-grain weight (*P<* 0.05). In contrast, no significant differences in the effective panicle number were observed between treatments (**Table 6**). Take the results of the Wanmai 52in the wheat growing season from 2020 to 2021 as an example, compared with the control, the number of grains per spike decreased by 17.85%, 29.00%, and 36.05% in Wanmai 52, while the 1000-grain weight decreased by 17.91%, 22.87%, and 32.60%, respectively, following LTS treatment at booting of 2°C, 0°C and −2°C.

**Table 6:** Effect of LTS at booting on yield components of wheat.

Variety	Treatment	Number of productive ears		Grain number per ear		1000-grain weight (g)	
		2018–2019	2020–2021	2018–2019	2020–2021	2018–2019	2020–2021
Yannog19	control	30.33a ± 0.88	31.20a ± 1.16	46.50a ± 0.43	46.50a ± 1.26	45.51a ± 0.49	45.60a ± 0.12
	2°C	23.67a ± 0.88	22.20b ± 0.73	38.00a ± 0.45	36.83b ± 1.14	41.36b ± 0.21	39.88b ± 0.13
	0°C	21.67a ± 0.88	19.00c ± 1.05	34.83a ± 0.54	33.00c ± 0.58	38.34c ± 0.55	36.25c ± 0.34
	–2°C	23.00a ± 0.58	18.20c ± 0.66	33.17b ± 0.40	29.17d ± 0.75	35.64d ± 0.62	32.92d ± 0.59
Wanmai52	control	30.67a ± 1.45	31.40a ± 0.81	45.50a ± 0.76	44.83a ± 1.54	46.54a ± 0.53	46.57a ± 0.38
	2°C	21.33a ± 1.76	18.40b ± 0.93	37.67a ± 0.67	36.83b ± 1.11	36.39b ± 0.35	38.23b ± 0.27
	0°C	19.33a ± 0.67	16.80b ± 0.80	34.33a ± 0.42	31.83c ± 0.87	34.63c ± 0.60	35.92c ± 0.30
	–2°C	17.67a ± 0.67	14.20c ± 0.58	31.50b ± 0.76	28.67d ± 0.49	31.25d ± 0.32	31.39d ± 0.56

Control and 2, 0 and −2°C treatments are as in **Table 3**. Different lowercase letters indicate a significant difference between treatments according to Duncan’s multiple range test (P< 0.05). The number after “±”indicates the standard error.

## 4 Discussion

### 4.1 Effects of LTS at booting on the accumulation and transportation of photosynthetic dry matter

Leaf photosynthesis is the main source of wheat grain assimilates. The accumulation of assimilates in the wheat grains then determines the final yield (Plaut et al., 2004; Grzebisz and Potarzycki, 2022). Photosynthesis is also one of the most sensitive physiological processes to LTS, because it maintains the balance between the light energy absorbed by plant photosystem and the energy consumed by various metabolic pathways (Ensminger et al., 2006). For example, the *P*_n_ of wheat flag leaves was previously found to decrease significantly after LTS treatment at jointing (Liu et al., 2019), as well as at the anther separation stage (Zhang et al., 2022). In line with this, this study showed that LTS at booting caused a significant reduction in *P*_n_, *G*_s_ and *T*_r_ in the flag leaves at grain filling. This may have been due to damage of functional leaves under LTS, thereby inhibiting carbon assimilation, and reducing the photosynthetic capacity of the flag leaves (Mousavi et al., 2019).

In wheat, LTS causes stomata to close, which affects the absorption of CO_2_, reducing the supply of photosynthetic raw material (CO_2_), and thereby the photosynthetic rate, assimilate accumulation and dry matter accumulation (Agurla et al., 2018; Liu et al., 2019). Studies have also shown that LTS will affect the distribution and transfer of photosynthetic fixed carbon in plants, resulting in a significant decrease in ^13^C in leaves. (Zhang et al., 2021a). In this study, a significant decrease in ^13^C abundance in the flag leaves and grains was observed compared with the control, suggesting that LTS at booting hinders the synthesis of photosynthetic products and the transport of assimilates to the grains (Liu et al., 2019).

About 1/3 of the dry matter of wheat grains is obtained via transportation from vegetative organs before anthesis, while the remainder is composed of the accumulation of photosynthetic dry matter from functional leaves after anthesis (Sanchez-Bragado et al., 2014). Wheat grain yield therefore relies on the balance between the supply of photoassimilates for grain filling (source) and the ability of the grains to accumulate these photoassimilates (sink) (Herzog, 1982). LTS leads to imbalance in the source-sink relationship (Saleem et al., 2021), affecting the transport of dry matter from the source to the sink organs, which results in an insufficient nutrient supply to the ears, and an ultimate reduction in wheat yield (Ke et al., 2021). Moreover, with decreasing temperature, the contribution rate of pre-anthesis stored dry matter to grains increased gradually, while the contribution rate of post-anthesis accumulated dry matter to grains decreased gradually. This may be due to the fact that the damaged functional leaves could not carry out normal photosynthesis after LTS at booting, resulting in a reduction in the accumulation of post-anthesis photosynthetic dry matter, and thus, a reduction in the contribution rate of post-anthesis accumulated dry matter to grains.

### 4.2 Effect of LTS at booting on starch synthesis

Starch biosynthesis and deposition play a leading role in the process of starch accumulation in the wheat grains (Bahaji et al., 2014). A number of studies have revealed the role of Pho1 and dpe1 in starch synthesis. For example, Pho1 plays a key role in starch initiation by prolonging the chain length of initial primers (Satoh et al., 2008), as well as acting as an active protein at the beginning of starch biosynthesis in barley (Cuesta-Seijo et al., 2017). Meanwhile, the main function of Dpe1 during the synthesis of storage starch is to reshape the amylose and amylopectin molecules in cereal crops (van der Maarel and Leemhuis, 2013). Related studies have also shown that activity of Pho1 reaches a maximum at 12 d after anthesis in barley, while *Dpe1* expression is high in the very early stage of rice development, and at approximately 14 d after anthesis in wheat (Ohdan et al., 2005; Tickle et al., 2009; Cuesta-Seijo et al., 2017). In this study, activities of pho1 and dpe1 reached a peak at about 15 d after anthesis. Pho1 is greatly influenced by temperature change, and when temperatures drop below 20°C, starch synthesis in the mutant endosperm of rice Pho1 was found to be significantly damaged, resulting in atrophy of most seeds (Hwang et al., 2016). Changes in *Dpe1*gene expression not only affect the content of amylopectin, but they also alter its fine structure (Seung, 2020). In addition, genes related to the key enzymes of wheat starch synthesis (GBSSI, AGPase and Pho1) were found to be down-regulated under drought stress (Lu et al., 2019). Meanwhile, in this study, decreasing LTS treatment at booting caused a decrease in the activities of both starch synthesis-related enzymes, Pho1 and Dpe1, while the relative expression levels of *AGPase, GBSSI, SSSI, SSSII* and *Pho1* in the grains 20 d after anthesis also decreased. These results are thought to highlight the reason for the reduction in starch content in the wheat grains under LTS.

### 4.3 Effects of LTS at booting on the starch content of the grains

Starch is an important component of wheat grains, accounting for about 70% of the dry weight. It is composed of amylose and amylopectin, and its accumulation has a direct impact on wheat yield (Mukherjee et al., 2015; Cornejo-Ramírez et al., 2018; Bhalla and Garg, 2021). Under stress such as high temperatures and drought, the production of photosynthetic products is impaired, restricting the entry of photosynthetic carbon products into the sink organs (grains). The grain filling stage is shortened, and causing a reduction in starch accumulation (Barnabás et al., 2008; Dolferus et al., 2011). Abiotic stress also affects starch granule formation. For example, numerous cracks and holes were observed on the surface of A-type starch granules in wheat grains following acid rain and waterlogging stress (Cornejo-Ramírez et al., 2018), while a decrease in the proportion of B-type starch granules at maturity was observed after LTS treatment (Yu et al., 2020). High-temperature and drought stress were also found to cause a significant reduction in the proportion of A- and B-type starch granules in winter wheat grains (Zahra et al., 2021). Similarly, in this study, LTS at booting caused a significant reduction in the total soluble sugar content of the wheat grains at filling, and obvious equatorial grooves were observed on the surface of the A-type starch granules. Meanwhile, the number of B-type starch granules decreased, affecting the formation of grain starch.

The biosynthesis and accumulation of starch are affected by external environmental factors (Golfam et al., 2021). For example, studies have shown a decrease in the total starch content of wheat grains following low- and high-temperature stress during grain filling (Zhao et al., 2022), while drought stress decreased the content of total starch and amylose in the grains (Bala et al., 2018). Moreover, another study found that LTS during grain filling had no significant effect on the total starch content of rice, but increased the amylose content (Ahmed et al., 2008; Baek et al., 2018). Drought stress at grain filling was also found to result in an increase in starch and amylopectin in developing rice grains (Prathap et al., 2019). In this study, LTS at booting caused a significant reduction in the total starch content of the grains at filling, with the largest reduction at -2°C. This may have been due to the effect of impaired photosynthesis caused by LTS at booting on carbon accumulation and transportation, and the subsequent decrease in the content of starch synthesis sources (soluble sugars) in the grains, which in turn inhibits the synthesis and accumulation of starch.

### 4.4 Effects of LTS at booting on grain filling and yield

The most sensitive indicators of natural freezing damage are the number of grains per spike, followed by the number of ears and the 1000-grain weight (Wu et al., 2022). Studies have shown that climate changes, such as increases in CO_2_, temperature, and water stress, shorten the duration of wheat growth stages, resulting in reduced carbohydrate assimilation, and a subsequent reduction in the size of the ears, grain diameter and yield (Asif et al., 2019). Studies have also shown that LTS at booting affects final wheat yield by reducing the rate of grain filling (Zhang et al., 2021b), while high-temperature and drought stress resulted in a reduction in grain shrinkage (Vikender et al., 2016) and grain weight (Hlaváčová et al., 2018). LTS at booting was also found to cause a reduction in the number of grains per spike and grain weight (Ji et al., 2017). Similarly, in this study, LTS at booting shortened the filling time, reduced the mean and maximum filling rate, and significantly decreased the number of grains per spike and the 1000-grain weight.

**Figure 8:**
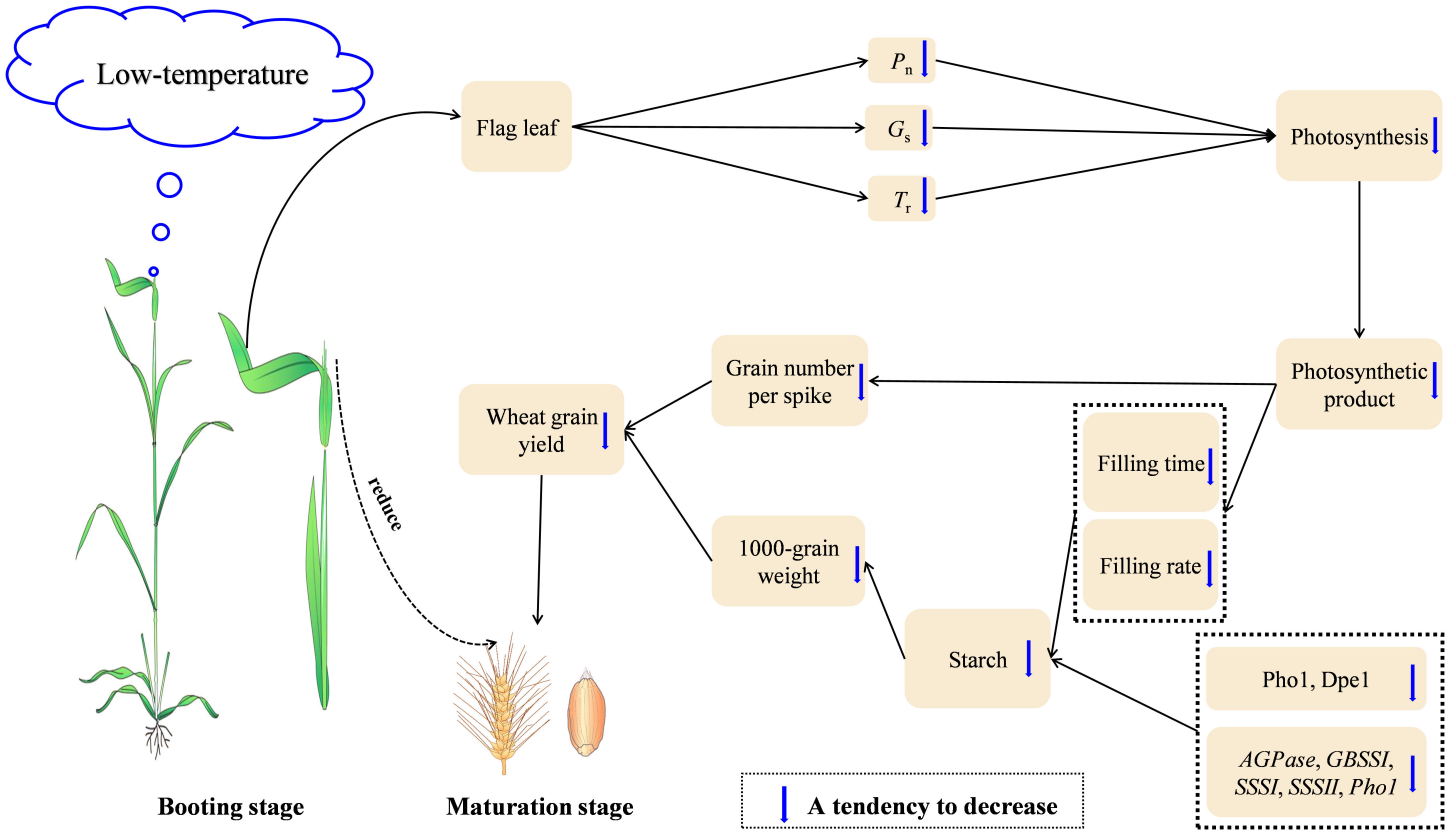
The proposed underlying mechanism of wheat yield reductions caused by short-term LTS at booting. LTS at booting reduces the transport and accumulation of dry matter, causing a reduction in yield. *P*_n_: net photosynthetic rate; *G*_s_: stomatal conductance; *T*_r_: transpiration rate.

Overall, LTS at booting damaged the photosynthetic capacity of the functional leaves, causing a decrease in dry matter accumulation and weakening the transport capacity of photosynthetic products to the grains, which led to the decrease of grain number per spike. The grain filling time was shortened, and the grain filling rate decreased. Moreover, the activity and relative expression of enzymes related to starch synthesis decreased, inhibiting the formation of grain starch granules and reducing the grain starch content. The number of grain number per spike and the 1000-grain weight decreased, leading to a reduction in wheat yield (**Figure 8**).

## 5 Conclusions

To summarize, LTS at booting reduced starch content and yield of wheat. *P*_n_, *G*_s_, and *T*_r_ of flag leaf decreased. The accumulation of photosynthates in the flag leaves and transportation to the grains decreased, causing a significant reduction in the contribution rate of photosynthetic products to the grains after anthesis and the distribution ratio of dry matter to the grains at maturity. The development of starch grains in the endosperm is also hindered. In the early stage of starch synthesis, the activities of key enzymes (Pho1 and Dpe1) decreased, and the relative expression levels of key enzymes (*AGPase, GBSSI, SSSI, SSSII*, and *Pho1*) decreased. The grain filling time and grain filling rate decreased, causing a reduction in the number of grains per spike and the 1000-grain weight.

## Data availability statement

The original contributions presented in the study are included in the article/supplementary material. Further inquiries can be directed to the corresponding author.

## Author contributions

WZ and ZH designed the experiment. WZ and AZ initiated statistical analysis and drafted the manuscript. AZ, QZ, RF, and YZ performed the experiments and determined related data. ZL, JZ and MZ contributed to the experiments proceeding and data interpretation. SM and YF helped in drafting the manuscript. All authors read and approved the final manuscript.

## References

[B1] Agurla S. Gahir S. Munemasa S. Murata Y. Raghavendra A. S. (2018). Mechanism of stomatal closure in plants exposed to drought and cold stress. Adv. Exp. Med. Biol. 1081, 215–232. doi: 10.1007/978-981-13-1244-1_12 30288712

[B2] Ahmed N. Maekawa M. Tetlow I. J. (2008). Effects of low temperature on grain filling, amylose content, and activity of starch biosynthesis enzymes in endosperm of basmati rice. Aust. J. Agric. Res. 59 (7), 599–604. doi: 10.1071/AR07340 25284759

[B3] Akdogan G. Kubota J. Kubo A. Takaha T. Kitamura S. (2011). Expression and characterization of rice disproportionating enzymes. J. Appl. Glycosci. 58 (3), 99–105. doi: 10.5458/jag.jag.JAG-2010_026

[B4] Asif M. Tunc C. E. Yazici M. A. Tutus Y. Rehman R. Rehman A.. (2019). Effect of predicted climate change on growth and yield performance of wheat under varied nitrogen and zinc supply. Plant Soil. 434, 231–244. doi: 10.1007/s11104-018-3808-1

[B5] Baek J. Jeong H. An S. Jeong J. Lee H. Yoon J.. (2018). Effects of low temperature during ripening on amylose content and enzyme activities associated with starch biosynthesis in rice endosperm. Korean J. Crop Sci. 63 (2), 86–97. doi: 10.7740/kjcs.2018.63.2.086

[B6] Bahaji A. Li J. Sánchez-López Á.M. Baroja-Fernández E. Muñoz F. J. Ovecka M.. (2014). Starch biosynthesis, its regulation and biotechnological approaches to improve crop yields. Biotechnol. Adv. 32 (1), 87–106. doi: 10.1016/j.biotechadv.2013.06.006 23827783

[B7] Bala S. Asthir B. Bains N. S. (2018). Heat and drought stress responses alter grain characteristics by impeding starch precursors of wheat. Indian J. Exp. Biol. 56, 565–572. Available at: https://nopr.niscpr.res.in/handle/123456789/44839.

[B8] Barnabás B. Jäger K. Fehér A. (2008). The effect of drought and heat stress on reproductive processes in cereals. Plant Cell Environ. 31 (1), 11–38. doi: 10.1111/j.1365-3040.2007.01727.x 17971069

[B9] Bhalla S. Garg N. (2021). Arbuscular mycorrhizae and silicon alleviate arsenic toxicity by enhancing soil nutrient availability, starch degradation and productivity in *Cajanus cajan* (L.) millsp. Mycorrhiza. 31 (6), 735–754. doi: 10.1007/s00572-021-01056-z 34669029

[B10] Clusters N. Ende W. V. D. Ceusters J. (2016). Exploration of sweet immunity to enhance abiotic stress tolerance in plants: lessons from CAM. Prog. Bot. 78, 145–166. doi: 10.1007/124_2016_1

[B11] Cornejo-Ramírez Y. I. Martínez-Cruz O. Del Toro-Sánchez C. L. Wong-Corral F. J. Borboa-Flores J. Cinco-Moroyoqui F. J. (2018). Características estructurales de almidones y sus propiedades funcionales. CYTA-J. Food. 16 (1), 1003–1017. doi: 10.1080/19476337.2018.1518343

[B12] Cromey M. G. Wright D. S. C. Boddington H. J. (1998). Effects of frost during grain filling on wheat yield and grain structure. New Zeal. J. Crop Hortic. 26 (4), 279–290. doi: 10.1080/01140671.1998.9514065

[B13] Cuesta-Seijo J. A. Ruzanski C. Krucewicz K. Meier J. Hägglund P. Svensson B.. (2017). Functional and structural characterization of plastidic starch phosphorylase during barley endosperm development. PloS One 12 (4), e0175488. doi: 10.1371/journal.pone.0175488 28407006 PMC5391026

[B14] Darroch B. A. Baker R. J. (1990). Grain filling in three spring wheat genotypes: statistical analysis. Crop Sci. 30 (3), 525–529. doi: 10.2135/cropsci1990.0011183X003000030009x

[B15] Dhakal A. (2021). Effect of drought stress and management in wheat–a review. Food Agribus. Manage. 2 (2), 62–66. doi: 10.26480/fabm.02.2021.62.66

[B16] Dolferus R. Ji X. Richards R. A. (2011). Abiotic stress and control of grain number in cereals. Plant Sci. 181 (4), 331–341. doi: 10.1016/j.plantsci.2011.05.015 21889038

[B17] Dong X. Zhang D. Liu J. Liu Q. Liu H. Tian L.. (2015). Plastidial disproportionating enzyme participates in starch synthesis in rice endosperm by transferring maltooligosyl groups from amylose and amylopectin to amylopectin. Plant Physiol. 169 (4), 2496–2512. doi: 10.1104/pp.15.01411 26471894 PMC4677918

[B18] Ensminger I. Busch F. Huner N. P. A. (2006). Photostasis and cold acclimation: sensing low temperature through photosynthesis. Physiol. Plant 126 (1), 28–44. doi: 10.1111/j.1399-3054.2006.00627.x

[B19] Golfam R. Kiarostami K. Lohrasebi T. Hasrak S. Razavi K. (2021). A review of drought stress on wheat (*Triticum aestivum* l.) starch. Farming Manage. 6 (1), 47–57. doi: 10.31830/2456-8724.2021.007

[B20] Grzebisz W. Potarzycki J. (2022). Effect of magnesium fertilization systems on grain yield formation by winter wheat (*Triticum aestivum* l.) during the grain-filling period. Agronomy. 12 (1), 12. doi: 10.3390/agronomy12010012

[B21] Gupta S. M. Agarwal A. Dev B. Kumar K. Prakash O. Arya M. C.. (2016). Assessment of photosynthetic potential of indoor plants under cold stress. Photosynthetica. 54, 138–142. doi: 10.1007/s11099-015-0173-7

[B22] Herzog H. (1982). Relation of source and sink during grain filling period in wheat and some aspects of its regulation. Physiol. Plant 56 (2), 155–160. doi: 10.1111/j.1399-3054.1982.tb00318.x

[B23] Hlaváčová M. Klem K. Rapantová B. Novotná K. Urban O. Hlavinka P.. (2018). Interactive effects of high temperature and drought stress during stem elongation, anthesis and early grain filling on the yield formation and photosynthesis of winter wheat. Field Crop Res. 221, 182–195. doi: 10.1016/j.fcr.2018.02.022

[B24] Hwang S. K. Koper K. Satoh H. Okita T. W. (2016). Rice endosperm starch phosphorylase (Pho1) assembles with disproportionating enzyme (Dpe1) to form a protein complex that enhances synthesis of malto-oligosaccharides. J. Biol. Chem. 291 (38), 19994–20007. doi: 10.1074/jbc.M116.735449 27502283 PMC5025686

[B25] Hwang S. K. Nishi A. Satoh H. Okita T. W. (2010). Rice endosperm-specific plastidial α-glucan phosphorylase is important for synthesis of short-chain malto-oligosaccharides. Arch. Biochem. Biophys. 495 (1), 82–92. doi: 10.1016/j.abb.2009.12.023 20045390

[B26] Ji H. Xiao L. Xia Y. Song H. Liu B. Tang L.. (2017). Effects of jointing and booting low temperature stresses on grain yield and yield components in wheat. Agric. For. Meteorol. 243, 33–42. doi: 10.1016/j.agrformet.2017.04.016

[B27] Kajla M. Yadav V. K. Khokhar J. Singh S. Chhokar R. S. Meena R. P.. (2015). Increase in wheat production through management of abiotic stresses: a review. J. Appl. Nat. Sci. 7 (2), 1070–1080. doi: 10.31018/jans.v7i2.733

[B28] Ke Y. Chen X. Zhang L. Zhang Y. Xu H. Muhammad A. H.. (2021). Effects of low temperature stress at anther connective stage on dry matter accumulation, translocation and distribution and grain yield of wheat. J. Anhui Agric. Univ. 48 (5), 701–706. doi: 10.13610/j.cnki.1672-352x.20211105.010

[B29] Kreslavski V. D. Zorina A. A. Los D. A. Fomina I. R. Allakhverdiev S. I. (2013). “Molecular mechanisms of stress resistance of photosynthetic machinery”, in Molecular stress physiology of plants. Eds. Rout G. R. Das A. B. (New Delhi, India: Springer), 21–51. doi: 10.1007/978-81-322-0807-5_2

[B30] Kumar R. Singh P. C. Singh S. (2018). A review report: low temperature stress for crop production. Int. J. Pure Appl. Biosci. 6 (2), 575–598. doi: 10.18782/2320-7051.3031

[B31] Liu L. Ji H. An J. Shi K. Ma J. Liu B.. (2019). Response of biomass accumulation in wheat to low-temperature stress at jointing and booting stages. Environ. Exp. Bot. 157, 46–57. doi: 10.1016/j.envexpbot.2018.09.026

[B32] Lu H. Hu Y. Wang C. Liu W. Ma G. Han Q.. (2019). Effects of high temperature and drought stress on the expression of gene encoding enzymes and the activity of key enzymes involved in starch biosynthesis in wheat grains. Front. Plant Sci. 10. doi: 10.3389/fpls.2019.01414 PMC6863091 31798603

[B33] Mousavi S. Regni L. Bocchini M. Mariotti R. Cultrera N. G. M. Mancuso S.. (2019). Physiological, epigenetic and genetic regulation in some olive cultivars under salt stress. Sci. Rep. 9 (1), 1–17. doi: 10.1038/s41598-018-37496-5 30705308 PMC6355907

[B34] Mukherjee S. Liu A. Deol K. K. Kulichikhin K. Stasolla C. Brûlé-Babel A.. (2015). Transcriptional coordination and abscisic acid mediated regulation of sucrose transport and sucrose-to-starch metabolism related genes during grain filling in wheat (*Triticum aestivum* l.). Plant Sci. 240, 143–160. doi: 10.1016/j.plantsci.2015.09.010 26475195

[B35] Obembe O. S. Hendricks N. P. Tack J. (2021). Decreased wheat production in the USA from climate change driven by yield losses rather than crop abandonment. PloS One 16 (6), e0252067. doi: 10.1371/journal.pone.0252067 34138898 PMC8211167

[B36] Ohdan T. Francisco Jr. P.B. Sawada T. Hirose T. Terao T. Satoh H.. (2005). Expression profiling of genes involved in starch synthesis in sink and source organs of rice. J. Exp. Bot. 56 (422), 3229–3244. doi: 10.1093/jxb/eri292 16275672

[B37] Palta J. A. Kobata T. Turner N. C. Fillery I. R. (1994). Remobilization of carbon and nitrogen in wheat as influenced by postanthesis water deficits. Crop Sci. 34 (1), 118–124. doi: 10.2135/cropsci1994.0011183X003400010021x

[B38] Panigrahi R. Kariali E. Panda B. B. Lafarge T. Mohapatra P. K. (2019). Corrigendum to: controlling the trade-off between spikelet number and grain filling: the hierarchy of starch synthesis in spikelets of rice panicle in relation to hormone dynamics. f. Funct. Plant Biol. 46 (6), 595–595. doi: 10.1071/FP18153_CO 32172735

[B39] Plaut Z. Butow B. J. Blumenthal C. S. Wrigley C. W. (2004). Transport of dry matter into developing wheat kernels and its contribution to grain yield under post-anthesis water deficit and elevated temperature. Field Crop Res. 86 (2–3), 185–198. doi: 10.1016/j.fcr.2003.08.005

[B40] Prathap V. Kishwar A. Archana S. Chandrapal V. Veda K. Viswanathan C.. (2019). Starch accumulation in rice grains subjected to drought during grain filling stage. Plant Physiol. Biochem. 142, 440–451. doi: 10.1016/j.plaphy.2019.07.027 31419646

[B41] Saleem M. Fariduddin Q. Janda T. (2021). Multifaceted role of salicylic acid in combating cold stress in plants: a review. J. Plant Growth Regul. 40, 464–485. doi: 10.1007/s00344-020-10152-x

[B42] Sanchez-Bragado R. Molero G. Reynolds M. P. Araus J. L. (2014). Relative contribution of shoot and ear photosynthesis to grain filling in wheat under good agronomical conditions assessed by differential organ δ^13^C. J. Exp. Bot. 65 (18), 5401–5413. doi: 10.1093/jxb/eru298 25053645 PMC4157716

[B43] Satoh H. Shibahara K. Tokunaga T. Nishi A. Tasaki M. Hwang S.. (2008). Mutation of the plastidial α-glucan phosphorylase gene in rice affects the synthesis and structure of starch in the endosperm. Plant Cell. 20 (7), 1833–1849. doi: 10.1105/tpc.107.054007 18621947 PMC2518224

[B44] Seung D. (2020). Amylose in starch: towards an understanding of biosynthesis, structure and function. New Phytol. 228 (5), 1490–1504. doi: 10.1111/nph.16858 32767769

[B45] Shahryar N. Maali-Amiri R. (2016). Metabolic acclimation of tetraploid and hexaploid wheats by cold stress-induced carbohydrate accumulation. J. Plant Physiol. 204, 44–53. doi: 10.1016/j.jplph.2016.06.019 27500556

[B46] Tickle P. Burrell M. M. Coates S. A. Emes M. J. Tetlow I. J. Bowsher C. G. (2009). Characterization of plastidial starch phosphorylase in *Triticum aestivum* l. endosperm. J. Plant Physiol. 166 (14), 1465–1478. doi: 10.1016/j.jplph.2009.05.004 19524321

[B47] Trnka M. Rötter R. P. Ruiz-Ramos M. Kersebaum K. C. Olesen J. E. Žalud Z.. (2014). Adverse weather conditions for European wheat production will become more frequent with climate change. Nat. Clim. Change 4, 637–643. doi: 10.1038/nclimate2242

[B48] van der Maarel M. J. E. C. Leemhuis H. (2013). Starch modification with microbial alpha-glucanotransferase enzymes. Carbohydr. Polym. 93 (1), 116–121. doi: 10.1016/j.carbpol.2012.01.065 23465909

[B49] Vikender K. Singh S. Behl R. K. (2016). Heat and drought tolerance in wheat: integration of physiological and genetic platforms for better performance under stress. Ekin J. 2 (1), 1–14. Available at: https://dergipark.org.tr/en/pub/ekinjournal/issue/22787/243200.

[B50] Vinje M. A. Walling J. G. Henson C. A. Duke S. H. (2022). Temporal expression analysis of barley disproportionating enzyme 1 (*DPE1*) during grain development and malting. J. Am. Soc Brew. Chem. 1–8. doi: 10.1080/03610470.2022.2104060

[B51] Wu Y. Liu B. Gong Z. Hu X. Ma J. Ren D.. (2022). Predicting yield loss in winter wheat due to frost damage during stem elongation in the central area of Huang-huai plain in China. Field Crop Res. 276, 108399. doi: 10.1016/j.fcr.2021.108399

[B52] Xiao L. Asseng S. Wang X. Xia J. Zhang P. Liu L.. (2022). Simulating the effects of low-temperature stress on wheat biomass growth and yield. Agric. For. Meteorol. 326, 109191. doi: 10.1016/j.agrformet.2022.109191

[B53] Xiao L. Liu L. Asseng S. Xia Y. Tang L. Liu B.. (2018). Estimating spring frost and its impact on yield across winter wheat in China. Agric. For. Meteorol. 260, 154–164. doi: 10.1016/j.agrformet.2018.06.006

[B54] Yordanova R. Popova L. (2007). Effect of exogenous treatment with salicylic acid on photosynthetic activity and antioxidant capacity of chilled wheat plants. Gen. Appl. Plant Physiol. 33 (3–4), 155–170. Available at: http://obzor.bio21.bas.bg/ipp/gapbfiles/v-33/07_3-4_155-170.pdf.

[B55] Yu X. Hao D. Yang J. Ran L. Zang Y. Xiong F. (2020). Effects of low temperature at stem elongation stage on the development, morphology, and physicochemical properties of wheat starch. PeerJ. 8, e9672. doi: 10.7717/peerj.9672

[B56] Zahra N. Wahid A. Hafeez M. B. Ullah A. Siddique K. H. M. Farooq M. (2021). Grain development in wheat under combined heat and drought stress: plant responses and management. Environ. Exp. Bot. 188, 104517. doi: 10.1016/j.envexpbot.2021.104517

[B57] Zhang Y. Liu L. Chen X. Li J. (2022). Effects of low-temperature stress during the anther differentiation period on winter wheat photosynthetic performance and spike-setting characteristics. Plants. 11 (3), 389. doi: 10.3390/plants11030389 35161371 PMC8840500

[B58] Zhang H. Liu H. Li Z. Ding H. Xue Z. Zhao F.. (2021a). Effects of temperature and nitrogen application on photosynthetic characteristics and the absorption and distribution of carbon and nitrogen in apple plants. Photosynthetica. 59, 538–546. doi: 10.32615/ps.2021.044

[B59] Zhang W. Zhao Y. Li L. Xu X. Yang L. Luo Z.. (2021b). The effects of short-term exposure to low temperatures during the booting stage on starch synthesis and yields in wheat grain. Front. Plant Sci. 12. doi: 10.3389/fpls.2021.684784 PMC8300962 34305982

[B60] Zhao K. Tao Y. Liu M. Yang D. Zhu M. Ding J.. (2022). Does temporary heat stress or low temperature stress similarly affect yield, starch, and protein of winter wheat grain during grain filling? J. Cereal Sci. 103, 103408. doi: 10.1016/j.jcs.2021.103408

